# Effects of Low-Dose Bisphenol A on DNA Damage and Proliferation of Breast Cells: The Role of c-Myc

**DOI:** 10.1289/ehp.1409199

**Published:** 2015-05-01

**Authors:** Daniella Pfeifer, Young Min Chung, Mickey C-T. Hu

**Affiliations:** Division of Gynecologic Oncology, Department of Obstetrics and Gynecology, Stanford University School of Medicine, Stanford, California, USA

## Abstract

**Background:**

Humans are exposed to low-dose bisphenol A (BPA) through plastic consumer products and dental sealants containing BPA. Although a number of studies have investigated the mammary gland effects after high-dose BPA exposure, the study findings differ. Furthermore, there has been a lack of mechanistic studies.

**Objective:**

The objective of this study was to investigate the effect and the mechanism of low-dose BPA in mammary gland cells.

**Methods:**

We evaluated DNA damage following BPA exposure using the comet assay and immunofluorescence staining, and used cell counting and three-dimensional cultures to evaluate effects on proliferation. We examined the expressions of markers of DNA damage and cell-cycle regulators by immunoblotting and performed siRNA-mediated gene silencing to determine the role of c-Myc in regulating BPA’s effects.

**Results:**

Low-dose BPA significantly promoted DNA damage, up-regulated c-Myc and other cell-cycle regulatory proteins, and induced proliferation in parallel in estrogen receptor-α (ERα)-negative mammary cells. Silencing c-Myc diminished these BPA-induced cellular events, suggesting that c-Myc is essential for regulating effects of BPA on DNA damage and proliferation in mammary cells.

**Conclusions:**

Low-dose BPA exerted c-Myc–dependent genotoxic and mitogenic effects on ERα-negative mammary cells. These findings provide significant evidence of adverse effects of low-dose BPA on mammary cells.

**Citation:**

Pfeifer D, Chung YM, Hu MC. 2015. Effects of low-dose bisphenol A on DNA damage and proliferation of breast cells: the role of c-Myc. Environ Health Perspect 123:1271–1279; http://dx.doi.org/10.1289/ehp.1409199

## Introduction

Bisphenol A (BPA) is widely found in a number of household products, including food and water containers, linings of metal food and beverage cans, and in dental fillings (Rochester 2013). BPA is routinely detected in human urine, blood, and breast milk due to low but continuous exposure (Lassen et al. 2014; Peretz et al. 2014; Yi et al. 2013). Nine of 10 Americans tested positive with BPA in random urine samples (Lassen et al. 2014). In a study measuring BPA in breast adipose tissue, more than half of the examined samples had detectable BPA levels (Fenichel et al. 2013).

BPA has no structural homology with 17β-estradiol (E2), but because it is similar to diethylstilbestrol (DES), the synthetic estrogen known to cause cancer (Kurosawa et al. 2002), BPA has long been suspected to be able to induce carcinogenesis (Keri et al. 2007). Rodents exposed to environmentally relevant BPA levels around the time of birth showed characteristics associated with an increased risk for developing breast cancer (Ayyanan et al. 2011; Muñoz-de-Toro et al. 2005). However, the mechanism by which BPA exerts its biological actions is still obscure. Although the effects of high-dose BPA on proliferation in estrogen receptor-α (ERα)-positive cells have been studied (Olsen et al. 2003; Samuelsen et al. 2001), the molecular mechanisms underlying low-dose BPA–mediated regulation of proliferation and DNA damage in ERα-negative breast cells are largely unknown. Moreover, considering that a majority of adult human breast tissues expresses low levels of ERα (Boyd et al. 1996), nonestrogenic mechanisms of action for BPA need to be elucidated.

One mechanism that has been suggested to explain BPA toxicity in DNA damage is the production of reactive oxygen species (ROS) (Kovacic 2010). However, it remains unclear whether BPA at low (nanomolar) doses can induce ROS and DNA damage in ERα-negative mammary cells. DNA double-strand breaks (DSB) are the most harmful damage to genome integrity among the various types of damage to DNA (Rothkamm and Löbrich 2003). Ataxia-telangiectasia mutated (ATM) is activated through its autophosphorylation at Ser-1981 (ATM-pS1981). This activation of ATM induces phosphorylation of its downstream targets histone H2A variant H2AX at Ser-139 (γ-H2AX), another marker for DSB and DNA damage (Bakkenist and Kastan 2004). In addition to the ATM-mediated DNA damage response, it has been found that up-regulation of oncogenic c-Myc induces ROS and promotes DNA damage (Vafa et al. 2002). Moreover, c-Myc up-regulation suppresses repair of DNA DSB (Li et al. 2012) that can lead to oncogene-induced genetic instability, which is an evolving hallmark of cancer (Negrini et al. 2010). In parallel, deregulated c-Myc can also debilitate p53-mediated cell cycle arrest (Vafa et al. 2002) and promote proliferative signaling, which is a hallmark of cancer (Hanahan and Weinberg 2011). However, it is unknown whether low-dose BPA can act as a DNA damaging agent to induce phosphorylation of these DNA damage-associated proteins and to promote up-regulation of c-Myc in mammary cells.

Studies on the mitogenic, apoptotic, and transcriptional properties of BPA are inconsistent (Dairkee et al. 2008; Diel et al. 2002). The inconsistency has been attributed to wide variation in doses and the high (micromolar range) doses that are often used (LaPensee et al. 2009). Studies of cellular effects on mammary cells after low, environmentally relevant doses of BPA are needed. A study conducted by the Centers for Disease Control and Prevention detected BPA in 95% of urine samples from a reference population of 394 American adults (Calafat et al. 2005). That study reported average levels of total BPA in male and female urine of 1.63 and 1.12 ng/mL, respectively, suggesting lower exposures in women compared with men. Based on > 80 published human biomonitoring studies, it has been estimated that unconjugated BPA was routinely detected in blood in the range of nanograms per milliliter, which is similar to the range of conjugated BPA detected in the urine samples (Vandenberg et al. 2010). It has been estimated, based on measurable levels of BPA in body fluids and tissues, that the theoretical internal dose of the general population is 10–100 nM (Vandenberg et al. 2007). Therefore, we chose to study the effects of environmentally relevant doses of BPA in the nanomolar range (10–100 nM). In discussions on BPA safety, it is mentioned that evidence at the basic molecular level is often missing (Kovacic 2010). Therefore, we sought in this study to identify the molecular mechanisms mediating the effect of BPA on mammary cells.

In this study, we examined the effects of low-dose BPA exposure on breast cell lines at the cellular and the molecular levels to understand the mechanism(s) by which BPA leads to mammary gland effects. We also examined the ability of BPA to regulate the levels of proteins associated with DNA damage and proteins controlling proliferation in these cells at low doses. Here we show that low-dose BPA independently promotes DNA damage and proliferation and that c-Myc up-regulation is a key mechanism by which BPA exerts its DNA-damaging and proliferative effects on ERα-negative mammary cells.

## Materials and Methods

*Materials*. Materials and reagents used in these analyses are listed in Supplemental Material, “Chemicals and antibodies.”

*Cell lines and siRNA transfection.* The MCF10A (ERα-negative) and 184A1 (ERα-negative) human cell lines are immortalized benign and normal breast epithelial cell lines, respectively. The MCF7 (ERα-positive) and MDA-MB-231 (ERα-negative) cell lines originate from human breast epithelial adenocarcinomas. All of these cell lines were obtained from ATCC. Details regarding cell culture conditions are summarized in Supplemental Material, “Cell culture conditions.” For transfection with siRNA, specific siRNA against c-Myc (sc-29226) and control siRNA (sc-44231) were obtained from Santa Cruz Biotechnology. MCF10A and 184A1 cells were transfected with the c-Myc siRNA or control siRNA by using DharmaFECT 1 transfection reagent (Thermo Scientific) as described previously (Chung et al. 2012).

*Western blotting (immunoblotting)*. Cells were washed twice in phosphate-buffered saline (PBS) and then lysed with a lysis buffer containing protease inhibitors for 20 min on ice. Whole cell lysates were centrifuged at 10,000 × *g* for 15 min to remove cell debris. The protein concentration of lysates was determined with the Bio-Rad Protein Assay. Cell lysates (30 μg protein/each) were electrophoresed onto sodium dodecyl sulfate polyacrylamide electrophoresis gels. The separated proteins were transferred to nitrocellulose membranes (Bio-Rad) before blocking with 5% bovine serum albumin (BSA) in Tris-buffered saline (TBS) with 0.1% Tween-20 (Sigma). Membranes were incubated with primary antibody and then with horseradish peroxidase–conjugated secondary antibody in blocking buffer. The immunoblots were visualized on film with the West-Q chemiluminescence kit (GenDEPOT). β-actin represents the loading controls.

*Immunofluorescence*. Cells were seeded on round coverslips and were treated with ethanol (EtOH) or BPA 24 hr after seeding. The cells were washed in PBS, fixed in 4% paraformaldehyde for 15 min at 4°C, and washed in PBS again before permeabilization with PBS containling 0.1% Triton-X (Sigma). The coverslips were blocked in 1% BSA in permeabilization buffer for 1 hr at room temperature and incubated overnight with specific antibodies as described (Chung et al. 2012). After washing, the coverslips were incubated with an Alexa-488–conjugated secondary antibody for 1 hr, and the nuclei were stained with DAPI (4´,6-diamidino-2-phenylindole) solution (Sigma) for 10 min. After washing, the coverslips were mounted on glass slides and observed under a Leica-TCS-SP2 Laser Scanning Spectral Confocal Microscope. Images were acquired using Leica-SP2 software. A cell with more than five staining foci was considered positive.

*The comet assay*. To determine the level of DNA damage in 184A1, cells were treated with EtOH or BPA for 3 or 24 hr and then subjected to a comet assay to detect DNA damage and repair at the level of single cells under neutral conditions, as described previously (Tsai et al. 2008). Briefly, after treatment, cells were harvested and mixed with low-melting-temperature agarose. After lysis, electrophoresis was performed at 1 V/cm and 15 mA for 40 min. Slides were stained with SYBG Green dye for 10 min. One hundred randomly selected cells per sample were captured under a Zeiss fluorescent microscope, and digital fluorescent images were obtained using AxioVision software (version 4.8.2; Zeiss). The relative length and intensity of SYBR Green–stained DNA tails to heads is proportional to the amount of DNA damage present in the individual nuclei and is measured by Olive tail moment using TriTek CometScore software (TriTek Corp.).

*Analyses of intracellular ROS levels*. Cells were seeded on round coverslips in 12-well plates, treated for 2 or 24 hr with EtOH or BPA, and subsequently incubated with 50 μM DCF-DA (2´,7´-dichlorofluorescein diacetate) and MitoTracker (both from Invitrogen) at 37°C in the dark for 30 min. Cells were then fixed with 4% paraformaldehyde, washed three times with PBS, counterstained with DAPI, and again washed three times with PBS. The coverslips were mounted on glass slides and observed under a confocal microscope, and images were acquired as described above.

*Cell proliferation assays*. Cell proliferation was evaluated by the cell-counting assay and the MTT assay as described previously (Park et al. 2014). For the cell-counting assay, cells were seeded in a 6-well plate at 25,000 cells/well in five replicates for each treatment. Following treatment, the number of cells was counted in duplicate in a hematocytometer using 0.4% Trypan Blue. For the MTT assay, 2,000–4,000 cells were seeded into 96-well plates. At the end of the treatment, cells were incubated with 200 μL 2.5% 3-(4,5-dimethylthiazol-2-yl)-2,5-diphenyltetrazolium bromide (MTT; Sigma) for 2 hr, and the absorbance was determined colorimetrically at 560 nm using an ELISA plate reader. Cell proliferation was calculated as the ratio of the absorbance from treated samples compared with that of the control sample.

*Three-dimensional (3D) culture*. Cells were seeded in eight-well chamber slides (Lab-Tek™ II; Thermo Scientific) at 5,000 cells/well and treated with EtOH or BPA; 3D-spheroid assays were performed as described previously (Hu et al. 2014). The wells were first coated with 60 or 120 μL of ECM (extracellular matrix) gel from Engelbreth-Holm-Swarm murine sarcoma (Sigma). The cells were seeded on top of the gel layer in Dulbecco’s modiﬁed Eagle’s medium/F12 medium for MCF10A cells with 2.5% ECM gel and 5 ng/mL EGF. Cells were treated with EtOH, BPA, or E2 24 hr after initial seeding. Cells were grown for 14 days with the top layer of media replaced with new treated media every 4 days. Analysis of the spheroid size and calculation of the spheroid number were performed as described previously by Hu et al. (2014).

*Statistical analysis*. All data are expressed as mean ± SD at least three determinations. Differences between two groups were analyzed by two-sided unpaired Student’s *t*-tests when the variances were equal. All statistical analyses were performed using GraphPad Prism statistical software (version 6.05; GraphPad Software) or Excel (Microsoft Corp.). *p*-Values < 0.05 were considered statistically significant.

## Results

*Effects of low-dose BPA on H2AX and ATM phosphorylation and* γ*-H2AX and ATM-pS1981 positive breast cells.* Because phosphorylation of DNA damage–associated proteins (ATM and H2AX) is a major indicator of DSB, we examined whether a low dose (10 nM) of BPA induces the levels of ATM-pS1981 and γ-H2AX with time course experiments in 184A1, MCF10A, MCF7, and MDA-MB-231 breast cells. Using immunoblotting, we observed that both ATM-pS1981 and γ-H2AX were indeed up-regulated when cells were exposed to BPA at 10 nM (see Supplemental Material, Figure S1A–D).

**Figure 1:**
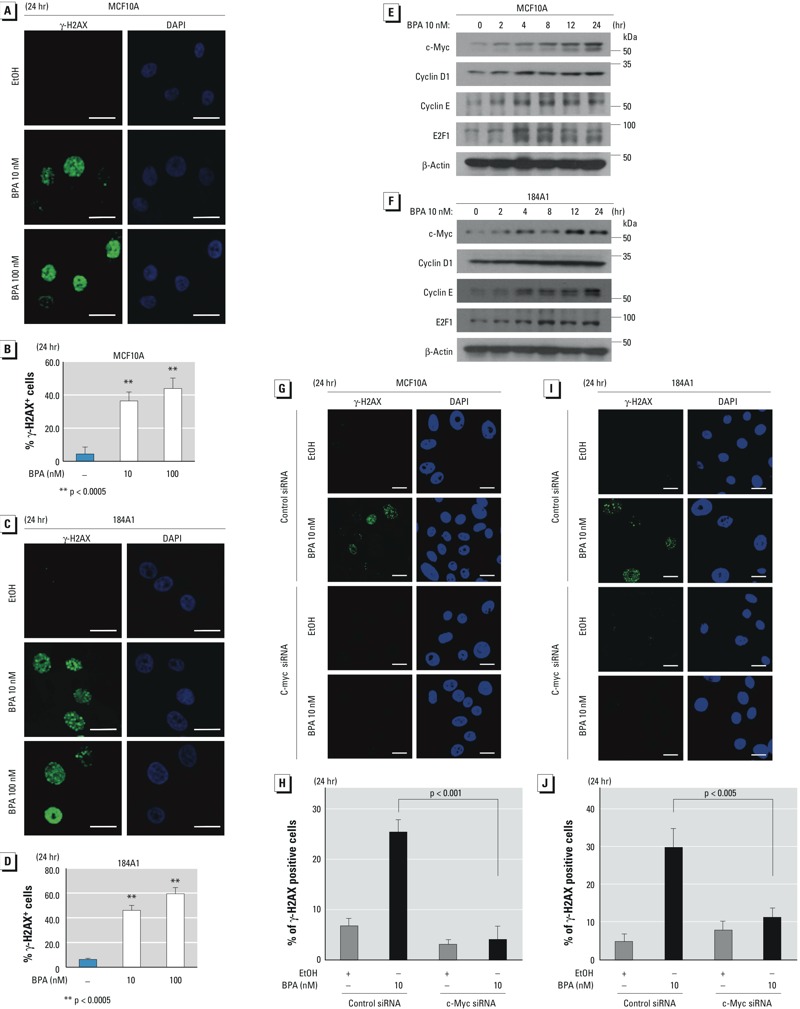
Low-dose BPA induces nuclear γ-H2AX levels through c-Myc. (*A,B*) MCF10A cells were treated with EtOH or BPA (10 or 100 nM) for 24 hr, and the levels and subcellular localization of endogenous γ-H2AX were detected using an anti-γ-H2AX antibody, followed by an Alexa-Fluor-488-conjugated secondary antibody; DAPI was used to visualize the nuclei. (*B*) An average of 150 of the stained cells was analyzed, and a histogram shows the percentage of cells with nuclei positive for γ-H2AX. (*C*,*D*) 184A1 cells were treated with EtOH or BPA for 24 hr, and the levels of γ-H2AX were detected; the histogram is displayed (*D*). MCF10A (*E*) and 184A1 (*F*) cells were treated with BPA (10 nM) for up to 24 hr, and total lysates from these cells were subjected to Western blotting using c‑Myc, cyclin D1, cyclin E, E2F‑1, and β-actin antibodies. MCF10A (*G*,*H*) and 184A1 (*I*,*J*) cells were transfected with c‑Myc–siRNA or control-siRNA for 48 hr, and the c‑Myc–knockdown and control cells were treated with EtOH or BPA for 24 hr; the levels of γ-H2AX were detected (*G,I*), and the histograms are shown (*H*,*J*). Bars = 20 μm (*A,C,G,I*). **p < 0.0005.

With immunofluorescence staining, foci of γ-H2AX can be seen at the points of DSB. To determine the effect of low doses of BPA on DNA damage, we analyzed the number of cells with γ-H2AX–positive nuclei after treatment with EtOH, 100 nM E2, 10 nM BPA, or 100 nM BPA. Compared with controls, all studied cell lines had a greater number of positive nuclei after BPA treatment for 3 hr (see Supplemental Material, Figure S1E,F) or 24 hr (Figure 1A–D; see also Supplemental Material, Figure S2A–D). The percentage of positive cells ranged from 40 to 60%, with the highest number of positive cells seen in the normal 184A1 cells. Phosphorylation of ATM at Ser-1981 was also investigated. Both MCF10A and MCF7 cells had a greater number of ATM-pS1981–positive nuclei after 24 hr treatment with BPA compared with the control (see Supplemental Material, Figure S2E–H). These data suggest that low-dose BPA may induce DSB in breast cells.

*Effects on c-Myc and other cell-cycle regulatory proteins*. One mechanism of DNA-damage induction in cells is through up-regulation of the c-Myc oncogenic protein (Vafa et al. 2002). Hence, we sought to determine whether low-dose BPA could up-regulate c-Myc in ERα-negative breast cells. By using immunoblotting, we observed that low-dose BPA (10 nM) significantly increased the level of c-Myc protein in a time-dependent manner in MCF10A and 184A1 cells (Figure 1E,F). Because c-Myc up-regulation can increase the cell proliferation rate (Schmidt 1999), we examined whether BPA could regulate the expression of other oncogenic cell-cycle regulatory proteins such as cyclin D1, cyclin E, and E2F1 in these cells under the same conditions. Similarly, our data show that low-dose BPA increased the levels of cyclin D1, cyclin E, and E2F1 in mammary cells (Figure 1E,F). However, the negative control EtOH did not alter the levels of these cell-cycle regulatory proteins in the treated cells (see Supplemental Material, Figure S3A). Similarly, our data show that BPA (10 nM) up-regulated the expression of these cell-cycle regulatory proteins in a time-dependent manner in MCF-7 [wild-type (wt)-p53] cells (see Supplemental Material, Figure S3B).

To test whether c-Myc is required for BPA-promoted increase of γ-H2AX, MCF10A and 184A1 cells were transfected with c-Myc siRNA or control siRNA for 48 hr, and c-Myc knockdown cells and control cells (see Supplemental Material, Figure S3C,D) were treated with EtOH or BPA for 24 hr and the levels of γ-H2AX were analyzed. Our results indicate that silencing c-Myc reduced BPA-mediated increase of γ-H2AX (Figure 1G–J), suggesting that c-Myc plays an essential role in BPA-induced DNA damage.

**Figure 2:**
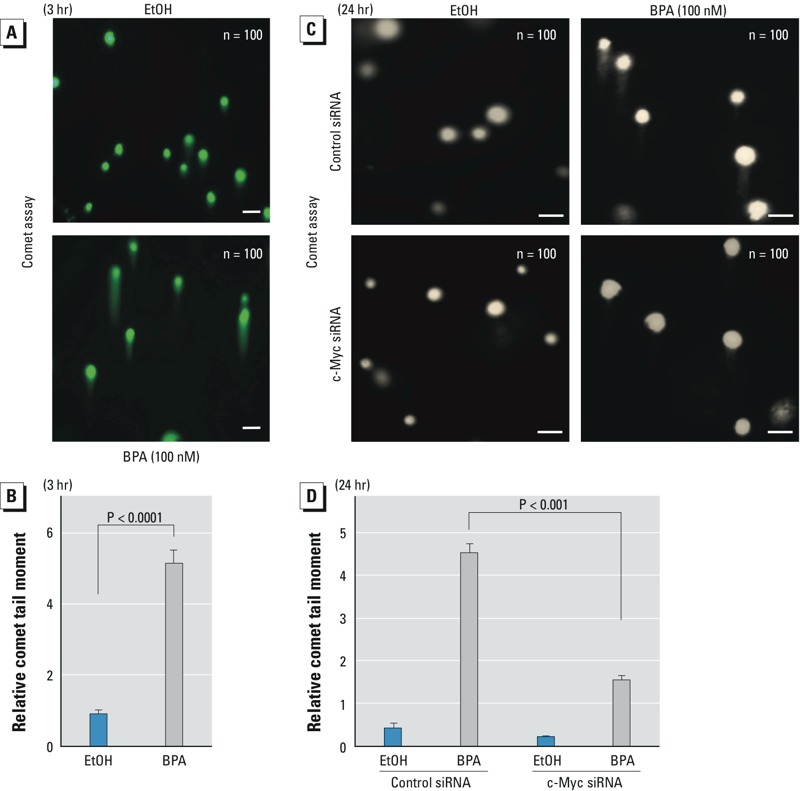
Low-dose BPA induces DNA DSB in normal mammary cells through c‑Myc. (*A,B*) 184A1 cells were treated with BPA or EtOH for 3 hr, and the comet assay was performed under neutral conditions. (*B*) The relative comet tail moments represent the averages of 100 cells in three independent experiments. (*C*,*D*) 184A1 cells were transfected with c‑Myc siRNA or control siRNA for 48 hr, the c‑Myc–knockdown and control cells were treated with EtOH or BPA for 24 hr, the comet assay was performed, and the relative comet tail moments are displayed (*B*). Bars = 40 μm.

*DNA damage*. Under neutral conditions, the comet assay directly correlates DNA DSB in individual cells with the relative tail moment, which is defined as the product of the distance between the head and the center of gravity of DNA in the tail and the percentage of DNA in the comet tail. The results of the comet assay illustrated staining of the DNA from individual from 184A1 cells after treatment with EtOH or 100 nM BPA. After 3-hr exposure to BPA, a significant increase of Olive tail moment was seen, showing that BPA induced rapid DSB in genomic DNA of normal mammary cells (Figure 2A,B) that is consistent with rapid formation of γ-H2AX foci (see Supplemental Material, Figure S1E,F).

To examine whether c-Myc is essential for BPA-induced DSB and whether both DSB and γ-H2AX foci take place at the same BPA exposure time (24 hr), c-Myc knockdown 184A1 cells and control cells were treated with EtOH or BPA for 24 hr and the levels of comet tail moment were analyzed. Our results show that silencing c-Myc significantly reduced BPA-mediated increase of the comet tail moment (Figure 2C,D), whose incubation time was consistent with that for γ-H2AX foci (Figure 1A–D). These results suggest that c-Myc is necessary for BPA-induced DSB.

**Figure 3:**
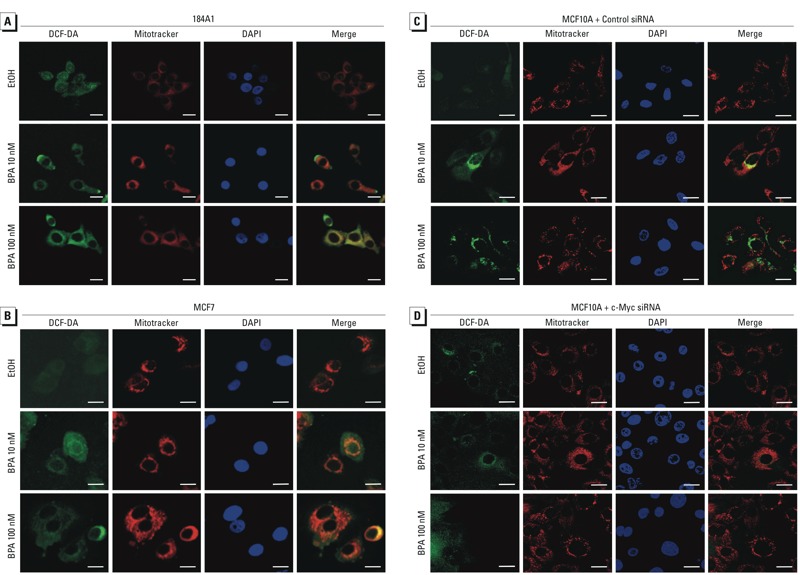
Low-dose BPA induces production of ROS in breast cells through c‑Myc. 184A1 (*A*) and MCF7 (*B*) cells were treated with BPA or EtOH for 2 hr; cells were stained with DCF-DA (10 μM) to show ROS-induced oxidation (green) and MitoTracker-Red (50 nM), a mitochondrial dye, and DAPI was used to visualize the nuclei. Merged images show the colocalization of mitochondria and ROS production. (*C*,*D*) MCF10A cells were transfected with control siRNA (*C*) or c‑Myc siRNA (*D*) for 48 hr, and c‑Myc–knockdown and control cells were treated with EtOH or BPA for 24 hr; ROS production was detected as described for (*A,B*). Bars = 20 μm.

*ROS production*. Because a major mechanism of DNA-damage induction is through oxidative stress, we utilized the fluorescent probe DCF-DA to determine whether low-dose BPA could induce ROS in breast cells. Figure 3A,B illustrates a rapid induction of ROS in 184A1 and MCF7 cells, respectively. Each was treated with 10 nM and 100 nM BPA, with an increase in staining intensity observed with higher dose. The ROS staining was localized to the mitochondria, as shown by the colocalization with Mitotracker staining, and incubation time is consistent with that for H2AX phosphorylation (γ-H2AX) (see Supplemental Material, Figure S4).

To assess whether c-Myc is required for BPA-induced ROS production, c-Myc knockdown MCF10A cells and control cells were treated with EtOH or BPA for 24 hr and the levels of production of ROS were analyzed. Our results indicate that silencing c-Myc abolished BPA-mediated ROS production (Figure 3C,D), and incubation time is consistent with that for γ-H2AX foci in Figure 1G–J. These results suggest that c-Myc is essential for BPA-promoted ROS production.

**Figure 4:**
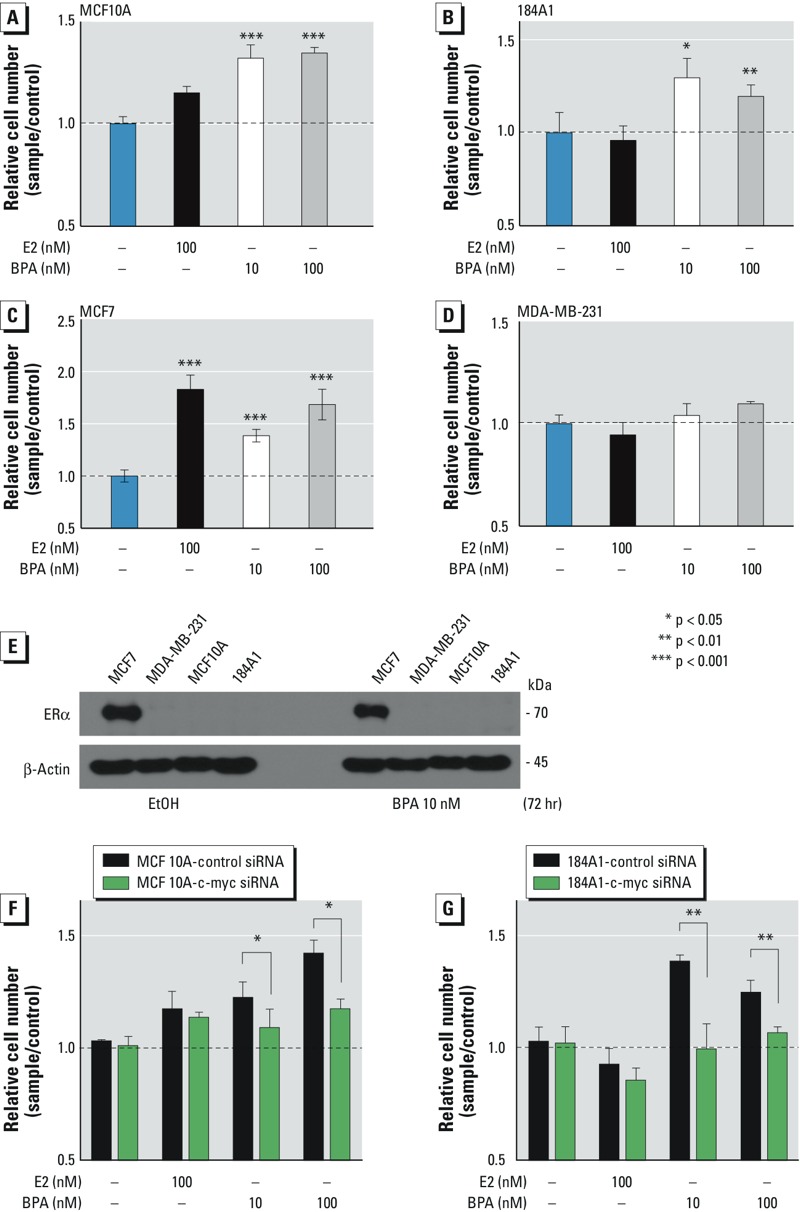
Low-dose BPA induces proliferation in wt-p53 breast cells through c‑Myc. Wt-p53 MCF10A (*A*), 184A1 (*B*), MCF-7 (*C*), and mutant-p53 MDA-MB-231 (*D*) cells were treated with EtOH (–), estradiol (E2), or BPA for 4 days; trypsinized; mixed with trypan blue; and counted in five replicates. The histograms show the relative number of cells (mean ± SD) in each sample compared with the control. (*E*) To show that the proliferation was not caused by an effect of BPA on up‑regulating ERα, Western blotting was performed using an anti-ERα antibody. Cells were treated with EtOH or 10 nM BPA for 72 hr, and the blot was stripped and reprobed for total proteins and β-actin. MCF10A (*F*) and 184A1 (*G*) cells were transfected with c‑Myc siRNA or control siRNA for 24 hr, and c‑Myc–knockdown and control cells were treated with EtOH or BPA for 48 hr; the relative number of cells are shown in the histograms.

*Proliferation of mammary cell lines*. An important question about the signaling mechanism is how low-dose BPA contributes to the increase of proliferation in ERα-negative noncancerous breast cells. c-Myc oncogenic protein can induce proliferation and transform mammary epithelial cells (Cowling et al. 2007). Thus, using the standard cell proliferation assays, we investigated whether low-dose BPA could induce proliferation in various breast cell lines. As shown in Figure 4A,B, low-dose BPA induced accelerated proliferation markedly in two ERα-negative wt-p53 mammary cell lines (MCF10A and 184A1). In contrast, E2 (100 nM) showed no significant effect on these cell lines. For comparison, we tested whether low-dose BPA could induce growth in breast cancer cell lines. As shown in Figure 4C, low-dose BPA potently augmented proliferation in wt-p53 MCF7 cells, whereas the effect of low-dose BPA on inducing proliferation was greatly diminished in the mutant-p53 cell line MDA-MB-231 (Figure 4D). Similar effects were seen with E2.

To examine whether the effect illustrated from BPA could be explained by an up-regulation of ERα, the expression of ERα was examined by immunoblotting in these cells after treatment with EtOH or BPA for 72 hr. Our data show that low-dose BPA exerts no effect on ERα expression in the treated cell lines (Figure 4E). These results confirm that low-dose BPA promotes proliferation in mammary cells in an ERα-independent manner.

To examine whether c-Myc is required for BPA-induced proliferation, c-Myc knockdown MCF10A and 184A1 cells and their control cells were treated with EtOH or BPA for 48 hr and the levels of proliferation were determined. Results show that silencing c-Myc significantly decreased BPA-mediated proliferation (Figure 4F,G), suggesting that c-Myc is essential for BPA-promoted proliferation.

**Figure 5:**
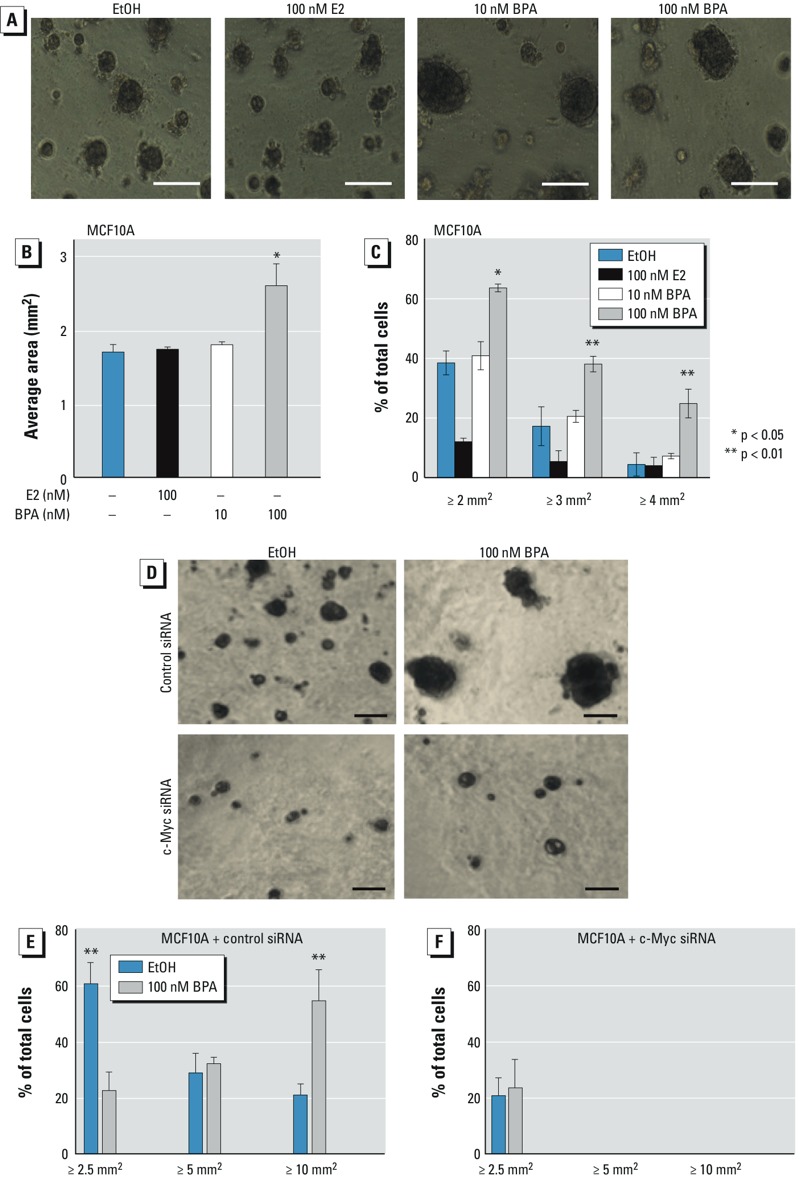
Low-dose BPA promotes spheroid proliferation in 3D cultures through c‑Myc. Cells were incubated for 10 days in 3D ECM-gel overlay cultures under treatment with EtOH, E2, or BPA. Cells were analyzed in a bright-field microscope and the area of each spheroid above a cutoff size was measured. (*A*) Phase-contrast/brightfield images at day 8; all images at the same magnification, resolution, and size. (*B*) The histogram shows the average size of spheroids (mean ± SD; *n* = 100; *p *= 0.004). (*C*) Percentage of spheroids that were two, three, and four times larger than the cutoff size is shown (mean ± SD of triplicate) as shown in (*B*). (*D*–*F*) MCF10A cells were transfected with c‑Myc siRNA or control siRNA for 24 hr, and c‑Myc–knockdown and control cells were treated with EtOH or BPA for 10 days in 3D cultures; images for spheroids are displayed in (*D*), and the histograms are presented in (*E,F*). Bars = 100 μm.

*Proliferation of mammary cells in 3D culture*. The mammary gland is composed of multiple cell types, and they are surrounded by an ECM. Together, they form complex interactions necessary for the mammary gland’s development and function (Polyak and Kalluri 2010). The conventional breast cell assays in 2D cell culture cannot mimic changes and interactions in authentic breast tissue and tumor microenvironments, whose distinctive 3D structures are critical for breast cancer development *in vivo*. Thus, to examine whether low-dose BPA would have the same proliferative effect on ERα-negative breast cells in a more tissue-like microenvironment, MCF10A cells were grown in an ECM gel in 3D spheroids. The size of spheroids was measured to determine whether the cells treated with low-dose BPA grew faster than the cells treated with ethanol (controls). Cells grown in 100 nM BPA had a higher median area and a higher percentage of spheroids two, three, and four times larger than the cutoff value compared with EtOH controls (Figure 5A–C). Treatment of these cells with 100 nM E2 seemed to have a negative effect on proliferation in 3D culture.

To test whether c-Myc is required for BPA-induced spheroid proliferation, c-Myc knockdown MCF10A cells and control cells were treated with EtOH or BPA and the levels of spheroid proliferation were determined. Our results indicate that silencing c-Myc significantly reduces BPA-mediated spheroid proliferation (Figure 5D–F), suggesting that c-Myc plays an essential role in BPA-promoted spheroid proliferation.

**Figure 6:**
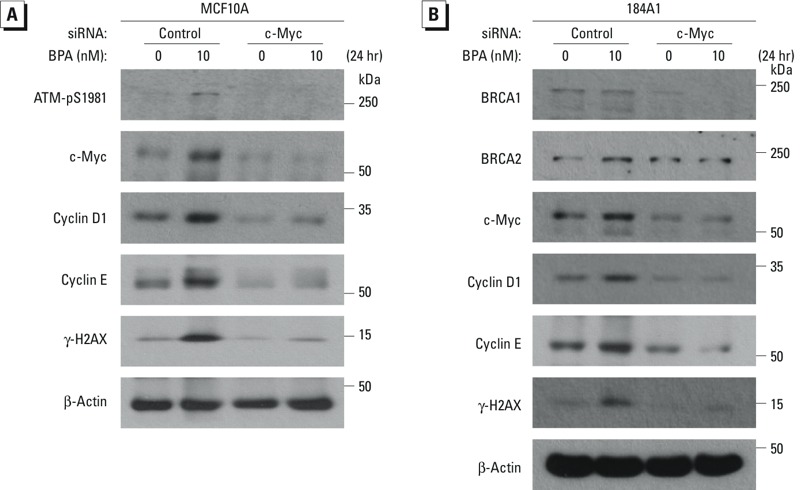
Silencing c‑Myc reduces the BPA-promoted expression of cell-cycle regulatory proteins. MCF10A (*A*) and 184A1 (*B*) cells were transfected with control siRNA or c‑Myc siRNA for 48 hr, and c‑Myc–knockdown and control cells were treated with BPA for 24 hr; whole lysates from these cells were subjected to Western blotting with ATM-pS1981, c‑Myc, cyclin D1, cyclin E, γ-H2AX, and β-actin antibodies in (*A*) and BRCA1, BRCA2, c‑Myc, cyclin D1, cyclin E, γ-H2AX, and β-actin antibodies in (*B*).

*Effect of c-Myc silencing on BPA-induced cell-cycle regulatory proteins*. To examine whether c-Myc is essential for BPA-mediated up-regulation of the expression of cell-cycle regulatory proteins, c-Myc knockdown MCF10A and 184A1 cells and their control cells were treated with EtOH or BPA and the levels of the specific cell-cycle proteins were analyzed. Data show that silencing c-Myc markedly decreased BPA-induced expression of the specific proteins (Figure 6A,B), suggesting that c-Myc is necessary for BPA-promoted oncogenic cell-cycle regulatory proteins. Collectively, our results suggest that low-dose BPA can induce the expression of oncogenic cell-cycle regulatory proteins leading to the increase of proliferation in ERα-negative mammary cells in a c-Myc–dependent manner.

## Discussion

In this study, we examined the effects of low concentrations of BPA (nanomolar doses) on DNA damage, the expression of oncogenic cell-cycle regulatory proteins, and the proliferation in normal (184A1), noncancerous (MCF10A), and cancerous (MCF7 and MDA-MB-231) breast cells. We have shown that *a*) BPA, at environmentally relevant doses, has a genotoxic effect on mammary cells *in vitro*; *b*) BPA up-regulates the expression of c-Myc and other crucial oncogenic cell-cycle regulatory proteins in these cells; *c*) BPA induces ROS in these cells; *d*) BPA has a mitogenic effect by increasing the rate of proliferation in wt-p53 breast cells *in vitro*; and *e*) c-Myc is required for all these cellular events induced by BPA. To our knowledge, this is the first study showing that low-dose BPA up-regulates c-Myc, which induces DNA damage and proliferation in ERα-negative mammary cells.

Previous studies have shown that high doses of BPA were cytotoxic but that exposure to BPA at low, noncytotoxic levels did not induce genotoxic effects (Audebert et al. 2011). Our results showed that BPA at noncytoxic levels did induce genotoxic effects. In the study by Audebert et al. (2011), cells were treated only for 24 hr, whereas in the present study cells were treated with BPA for up to 72 hr, and in some cell lines the DNA damaging effects were seen as late as 24–48 hr. Considering that BPA is thought to be a possible weak carcinogen (Cavalieri and Rogan 2010) and that the general population is exposed to a low chronic dose (Vandenberg et al. 2007), it is also of interest to study longer exposure times.

In a study of mice injected with ERα-dependent MCF7 cells and also treated with either high-dose BPA or a placebo, tumors were found in those treated with BPA (Weber Lozada and Keri 2011). In the present study, we examined the low-dose effect of BPA on DNA damage and proliferation in various mammary cell lines, and found that BPA induced DNA-damage markers in cells regardless of the ERα status. To our knowledge, this is the first study showing low-dose BPA (nanomolar doses) regulating nonestrogenic proliferation. We found that low-dose BPA increased proliferation in the ERα-negative cells, whereas E2 did not. In MCF-7 cells, however, both E2 and BPA increased proliferation. Although a higher dose of BPA usually displays a greater effect on proliferation in ER^+^ breast cancer cells, our BPA dose-dependent findings suggest that a higher dose of BPA may not excert a greater effect on proliferation in normal breast cells (184A1) (Figure 4B,G). These findings suggest that the dose-dependent effects of BPA on ER^+^ breast cancer cells may be distinct from those on ER^–^ normal mammary cells. Moreover, BPA did not induce ERα expression in ERα-negative breast cells. Our results confirm that the observed proliferative effects of BPA on these cells cannot be attributed to a potential up-regulation of ERα expression. Moreover, BPA has been shown to have the same antagonistic effect on anticancer drugs regardless of the ERα status of breast cells (LaPensee et al. 2009). Taken together, our data, as well as other studies, suggest that BPA can act on mammary cells through ERα-independent mechanisms.

High doses of BPA have been found to induce ERK1/2 (extracellular signal-regulated kinases) via GPR30 (G protein-coupled receptor 30) in breast cancer cells (Dong et al. 2011). In the present study, we found that when ERα-negative mammary cells were exposed to low-dose BPA, the expression of important oncogenic cell-cycle regulatory proteins, including c-Myc, cyclin D1, cyclin E, and E2F1, was significantly induced. It has been established that c-Myc increases proliferation rate and oncogenic transformation (Cowling et al. 2007; Schmidt 1999). Notably, up-regulating c-Myc also promotes DNA damage and induces ROS in cells (Vafa et al. 2002). These findings are consistent with our results that low-dose BPA exposures can induce c-Myc, increase ROS, and cause DNA damage, as well as promote proliferation in mammary cells. However, the possible mechanisms for BPA induction of ROS remain unclear. Ogrunc et al. (2014) reported that up-regulation of the Ras oncogene and its downstream effectors such as NADPH oxidases (NOXs) can induce ROS. Thus, it is possible that BPA-mediated up-regulation of the c-Myc oncogene may promote NOXs and increase ROS in breast cells.

Recently, some additional pathways have been suggested to be involved in the adverse effects of BPA exposure. For instance, while BPA exposure induces activation of the mTOR pathway (Akt1, RPS6, pRPS6, and 4EBP1), it reduces the p53 proapoptotic pathway (p53, p21WAF1, and BAX) in human breast epithelial cells, resulting in increased gene products that initiate proliferation (including proliferating cell nuclear antigen, cyclins, cyclin-dependent kinases, band phosphorylated pRb) (Dairkee et al. 2013). Thus, the effects of these additional pathways after BPA exposure can be tested to further build an understanding of the adverse outcome pathway for BPA exposure and mammary gland cellular effects.

In proliferation assays, our results indicate that low-dose BPA potently promoted proliferation in all three wt-p53 breast cell lines (Figure 4A–C), whereas the effect of low-dose BPA on inducing proliferation was decreased in mutant-p53 MDA-MB-231 cells (Figure 4D). These results suggest that the effect of BPA on inducing proliferation may depend on p53 function. In contrast, low-dose BPA potently induced DNA damage markers γ-H2AX and ATM-pS1981 in all four breast cell lines (i.e., MCF10A, 184A1, MCF7, and MDA-MB-231) (Figure 1A–D; see also Supplemental Material, Figures S1 and S2). These results suggest that BPA may induce DNA damage in MDA-MB-231 cells via a p53-independent mechanism.

Although low-dose BPA potently induced key DNA damage markers in MDA-MB-231 cells (see Supplemental Material, Figure S1D), low-dose BPA was unable to induce proliferation significantly in these cells (Figure 4D). These data strongly suggest that there is no causal relationship between DNA damage and proliferation. In fact, it has been documented that DNA damage induces cell cycle checkpoints, which lead to either cell cycle arrest for DNA break repair or induction of cellular apoptosis (Kastan and Bartek 2004; Zhou and Elledge 2000). Thus, DNA damage should not cause proliferation directly in normal breast cells, which have functional cell cycle checkpoints and DNA repair activity. Moreover, our data indicate that low-dose BPA considerably up-regulates the expression of c-Myc (Figures 1E,F and 6A,B) in mammary cells.

Although the ultimate goal is to extrapolate *in vitro* findings to human health, it is premature to do so. Further studies of the effect of low-dose BPA on mammary gland carcinogenesis and cancer development in rodent models are needed to validate the effect of low-dose BPA in an *in vivo* situation, which may contribute to new insight into this health problem.

Further investigation of the role of low-dose BPA in regulating global gene-expression changes and genome-wide epigenetic alternations in mammary cells would aid in assessing linkages between low-dose BPA exposure and cell proliferation, DNA damage, mammary gland transformation, and carcinogenesis. Understanding the molecular basis of potential effects of low-dose BPA on mammary carcinogenesis may suggest innovative preventive modalities to improve human health. Also, at least in theory, benefits of understanding the mechanism could extend beyond preventive measures targeted only at BPA to substances such as phthalates, parabens, and other environmental chemicals.

## Supplemental Material

(7 MB) PDF
