# Supplementary material for: Effects of Low-Dose Bisphenol A on DNA Damage and Proliferation of Breast Cells: The Role of c-Myc
Source: Environ Health Perspect. 2015 May 1;123(12):1271–9. doi: 10.1289/ehp.1409199 (PMC4671234; doi:10.1289/ehp.1409199)
Supplement: (7 MB) PDF [file ehp.1409199.s001.acco.pdf]

Environ Health Perspect
doi: 10.1289/ehp.1409199

Note to Readers: *EHP* strives to ensure that all journal content is accessible to all readers. However, some figures and Supplemental Material published in *EHP* articles may not conform to 508 standards due to the complexity of the information being presented. If you need assistance accessing journal content, please contact ehp508@niehs.nih.gov. Our staff will work with you to assess and meet your accessibility needs within 3 working days.

Supplemental Material

Effects of Low-Dose Bisphenol A on DNA Damage and Proliferation of Breast Cells: The Role of c-Myc

Daniella Pfeifer, Young Min Chung, and Mickey C-T. Hu

Table of Contents

Figure S1. A low dose of BPA induces DNA damage markers, γ-H2AX and ATM-pS1981, in breast cells, and low doses of BPA induce the levels of γ-H2AX in the nuclei of MCF10A cells. Cells were treated with BPA (10 nM) for 0, 12, 24, 48, and 72 hr in a time-course experiment. Total lysates from (A) human estrogen receptor-α (ERα)-negative normal-like 184A1, (B) ERα-negative noncancerous MCF10A, (C) ERα-positive cancerous MCF-7, and (D) ERα-negative cancerous MDA-MB-231 cells were subjected to Western blotting analysis using specific antibodies as indicated. The blots were stripped and re-probed for total proteins and loading control β-Actin. (E) MCF10A cells grown on coverslips were treated with ethanol (EtOH), 10 nM BPA or 100 nM BPA for 3 hr and fixed. The level and subcellular localization of endogenous γ-H2AX was detected using an antibody against γ-H2AX, followed by an Alexa Fluor-488-conjugated secondary antibody, and analyzed with a confocal fluorescence microscope. The dye 4'-6-Diamidino-2-phenylindole (DAPI) was used to visualize the nuclei. Scale bar: 20 μm. (F) An average of about 150 of the stained cells was analyzed and a histogram shows the percentage of cells with nuclei positive for γ-H2AX (≥ 5 foci).

Figure S2. Low doses of BPA induce the levels of γ-H2AX and ATM-pS1981 in the cell nuclei of noncancerous and cancerous breast cells. (A, B) ERα-positive MCF7 and (C, D) ERα-negative MDA-

MB-231 breast cancer cells grown on coverslips were treated with ethanol (EtOH), 10 nM BPA or 100 nM BPA for 24 hr and fixed. The level and subcellular localization of endogenous γ-H2AX was detected using an antibody (Ab) against γ-H2AX, followed by an Alexa Fluor-488-conjugated secondary Ab, and analyzed with a confocal fluorescence microscope. DAPI was used to visualize the nuclei. (B, D) An average of about 150 of the stained cells was analyzed and a histogram shows the percentage of cells with nuclei positive for γ-H2AX (≥ 5 foci). **, $P<0.0005$, compared with control. (E, F) ERα-negative noncancerous MCF10A and (G, H) ERα-positive cancerous MCF7 breast cells grown on coverslips were treated with the indicated agents for 24 hr. The level and subcellular localization of endogenous ATM-pS1981 was detected using an Ab against ATM-pS1981, followed by an Alexa Fluor-488-conjugated secondary Ab, and analyzed as described above. (F, H) An average of about 150 of the stained cells was analyzed and a histogram shows the percentage of cells with nuclei positive for ATM-pS1981 (≥ 5 foci). **, $P<0.0005$, compared with control. Scale bar: 20 μm.

Figure S3. The negative control does not alter the levels of c-Myc and other cell-cycle regulatory proteins, low-dose BPA increases the levels of these proteins in MCF-7 cells with wt-p53, and silencing c-Myc expression in noncancerous mammary cells abrogates BPA-induced c-Myc expression. (A) As controls, MCF10A and 184A1 cells were treated with ethanol (EtOH) for 0 or 24 hr, and total lysates from these cells were subjected to Western blotting (WB) using the indicated antibodies (Abs) and the blots were stripped and reprobed with an anti-β-Actin (loading control). (B) MCF-7 (wt-p53) cells were treated with BPA (10 nM) or negative control (0) for the indicated time course. Total lysates from these cells were subjected to WB analysis as described above. (C) MCF10A and 184A1 cells were transfected with control siRNA or c-Myc siRNA for 48 hr and total lysates of these cells were analyzed by WB with anti-c-Myc and anti-β-Actin Abs. (D) MCF10A cells grown on coverslips were transfected with control siRNA or c-Myc siRNA and treated with EtOH or BPA for 24 hr. The level and subcellular localization of endogenous c-Myc was detected using an anti-cMyc Ab, followed by an Alexa Fluor-594-conjugated secondary Ab, and analyzed with a confocal fluorescence microscope. DAPI was used to visualize the nuclei. Scale bar: 20 μm.

Figure S4. Low doses of BPA induce the levels of γ-H2AX and production of ROS concurrently in 184A1 cells after 2 hr exposure. 184A1 cells grown on coverslips were treated with negative control (EtOH) or BPA (10 or 100 nM) for 2 hr or 4 hr as indicated. Cells were fixed and stained with DCF-DA (10 μM) to show ROS-induced oxidation (green). The level and subcellular localization of endogenous γ-H2AX was detected using an antibody (Ab) against γ-H2AX, followed by an Alexa Fluor-594 (red)-conjugated secondary Ab, and analyzed with a confocal fluorescence microscope. DAPI was used to

visualize the nuclei. Merged images show the co-localization of γ-H2AX and ROS production. Scale bar: 20 μm.

Supplemental Materials and Methods

Chemicals and antibodies

Cell culture conditions

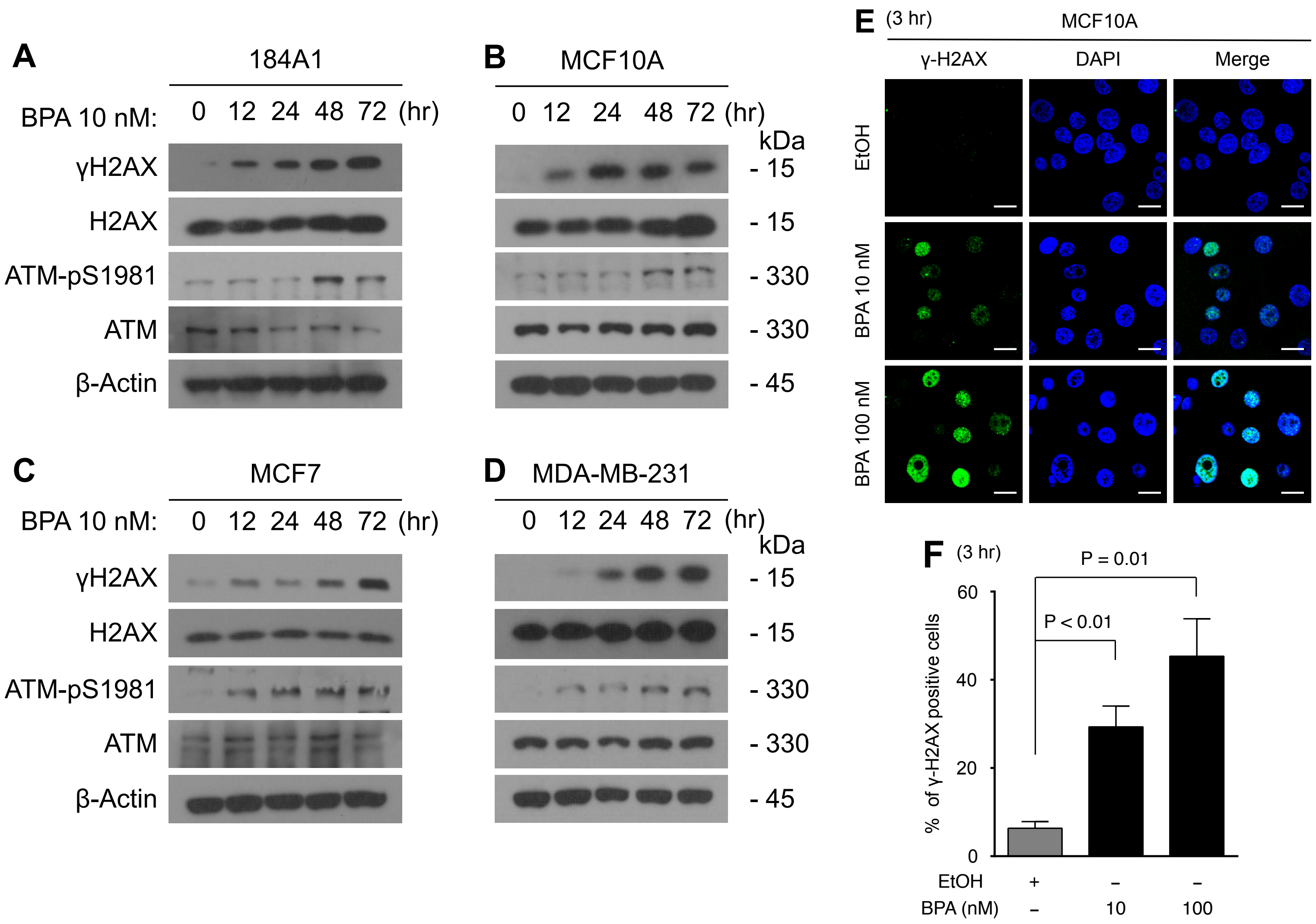

Figure S1. A low dose of BPA induces DNA damage markers, γ-H2AX and ATM-pS1981, in breast cells, and low doses of BPA induce the levels of γ-H2AX in the nuclei of MCF10A cells. Cells were treated with BPA (10 nM) for 0, 12, 24, 48, and 72 hr in a time-course experiment. Total lysates from (A) human estrogen receptor-α (ERα)-negative normal-like 184A1, (B) ERα-negative noncancerous MCF10A, (C) ERα-positive cancerous MCF-7, and (D) ERα-negative cancerous MDA-MB-231 cells were subjected to Western blotting analysis using specific antibodies as indicated. The blots were stripped and re-probed for total proteins and loading control β-Actin. (E) MCF10A cells grown on coverslips were treated with ethanol (EtOH), 10 nM BPA or 100 nM BPA for 3 hr and fixed. The level and subcellular localization of endogenous γ-H2AX was detected using an antibody against γ-H2AX, followed by an Alexa Fluor-488-conjugated secondary antibody, and analyzed with a confocal fluorescence microscope. The dye 4'-6-Diamidino-2-phenylindole (DAPI) was used to visualize the nuclei. Scale bar: 20 μm. (F) An average of about 150 of the stained cells was analyzed and a histogram shows the percentage of cells with nuclei positive for γ-H2AX (≥ 5 foci).

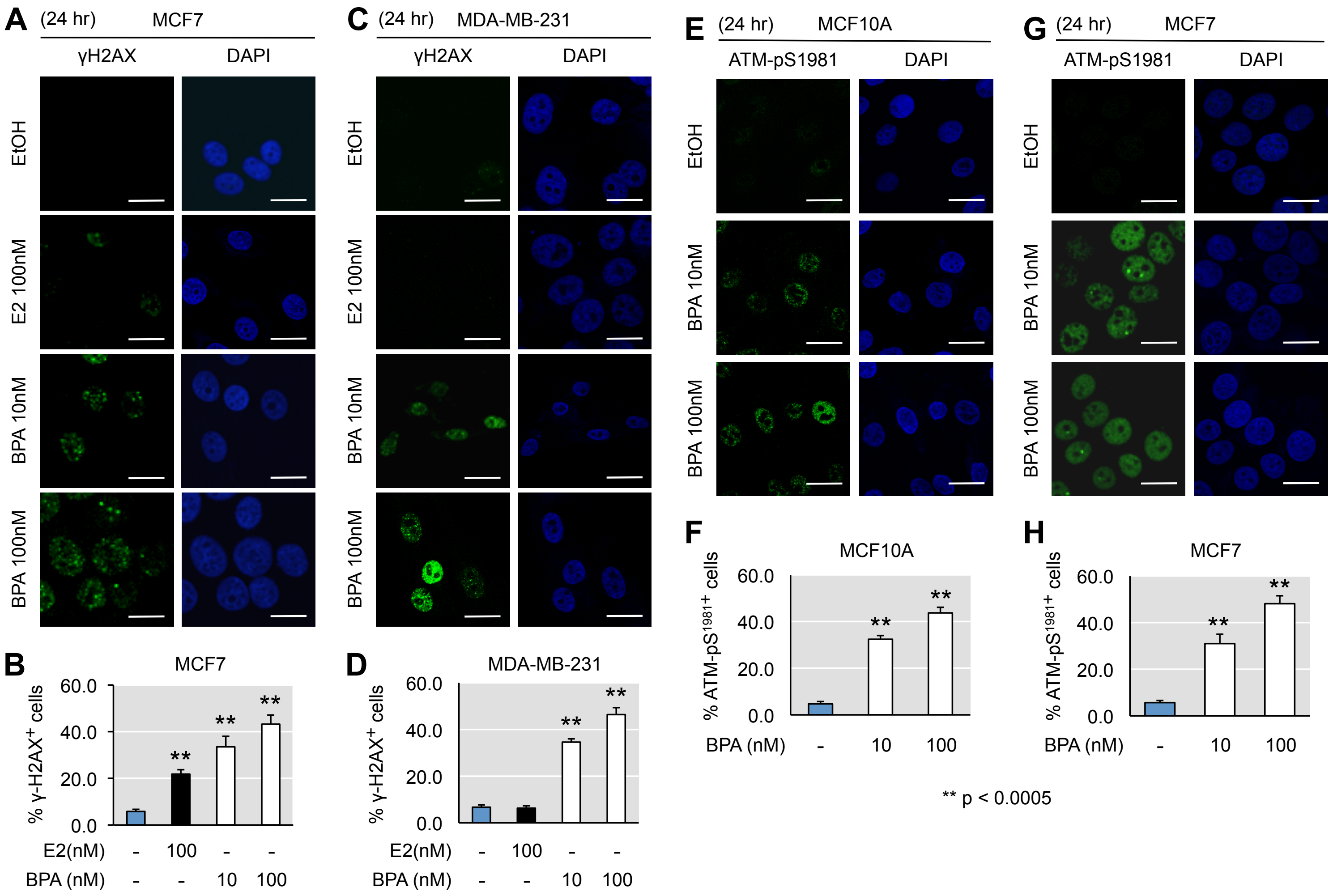

Figure S2. Low doses of BPA induce the levels of γ-H2AX and ATM-pS1981 in the cell nuclei of noncancerous and cancerous breast cells. (A, B) ERα-positive MCF7 and (C, D) ERα-negative MDA-MB-231 breast cancer cells grown on coverslips were treated with ethanol (EtOH), 10 nM BPA or 100 nM BPA for 24 hr and fixed. The level and subcellular localization of endogenous γ-H2AX was detected using an antibody (Ab) against γ-H2AX, followed by an Alexa Fluor-488-conjugated secondary Ab, and analyzed with a confocal fluorescence microscope. DAPI was used to visualize the nuclei. (B, D) An average of about 150 of the stained cells was analyzed and a histogram shows the percentage of cells with nuclei positive for γ-H2AX (≥ 5 foci). **, P<0.0005, compared with control. (E, F) ERα-negative noncancerous MCF10A and (G, H) ERα-positive cancerous MCF7 breast cells grown on coverslips were treated with the indicated agents for 24 hr. The level and subcellular localization of endogenous ATM-pS1981 was detected using an Ab against ATM-pS1981, followed by an Alexa Fluor-488-conjugated secondary Ab, and analyzed as described above. (F, H) An average of about 150 of the stained cells was analyzed and a histogram shows the percentage of cells with nuclei positive for ATM-pS1981 (≥ 5 foci). **, P<0.0005, compared with control. Scale bar: 20 μm.

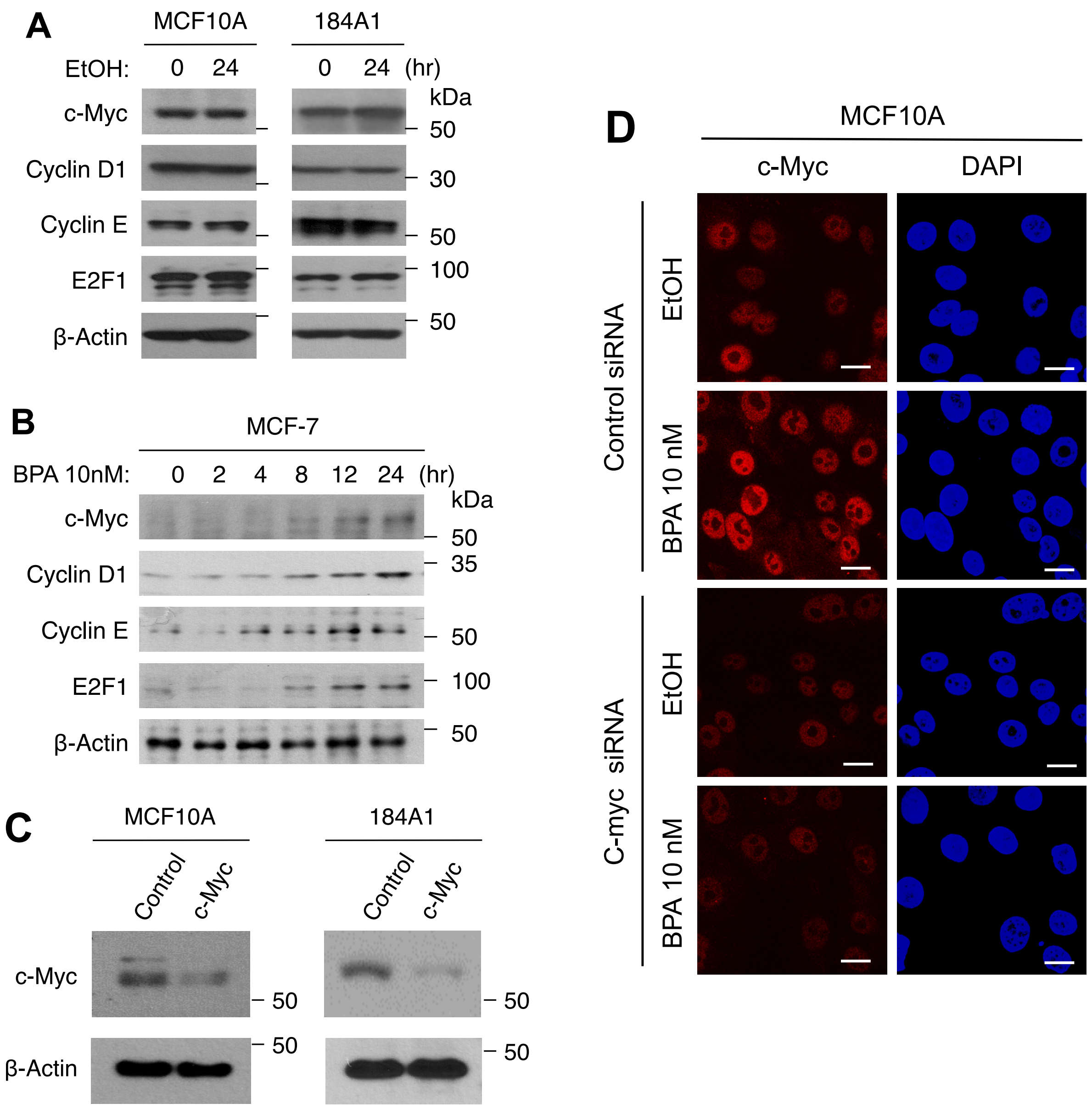

Figure S3. The negative control does not alter the levels of c-Myc and other cell-cycle regulatory proteins, low-dose BPA increases the levels of these proteins in MCF-7 cells with wt-p53, and silencing c-Myc expression in noncancerous mammary cells abrogates BPA-induced c-Myc expression. (A) As controls, MCF10A and 184A1 cells were treated with ethanol (EtOH) for 0 or 24 hr, and total lysates from these cells were subjected to Western blotting (WB) using the indicated antibodies (Abs) and the blots were stripped and reprobed with an anti-β-Actin (loading control). (B) MCF-7 (wt-p53) cells were treated with BPA (10 nM) or negative control (0) for the indicated time course. Total lysates from these cells were subjected to WB analysis as described above. (C) MCF10A and 184A1 cells were transfected with control siRNA or c-Myc siRNA for 48 hr and total lysates of these cells were analyzed by WB with anti-c-Myc and anti-β-Actin Abs. (D) MCF10A cells grown on coverslips were transfected with control siRNA or c-Myc siRNA and treated with EtOH or BPA for 24 hr. The level and subcellular localization of endogenous c-Myc was detected using an anti-cMyc Ab, followed by an Alexa Fluor-594-conjugated secondary Ab, and analyzed with a confocal fluorescence microscope. DAPI was used to visualize the nuclei. Scale bar: 20 μm.

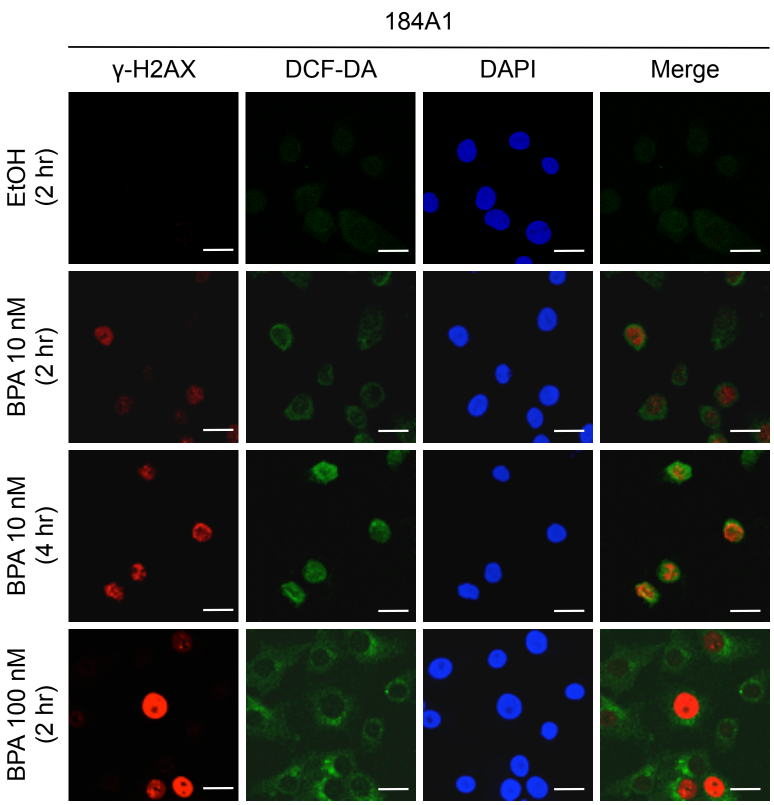

Figure S4. Low doses of BPA induce the levels of γ-H2AX and production of ROS concurrently in 184A1 cells after 2 hr exposure. 184A1 cells grown on coverslips were treated with negative control (EtOH) or BPA (10 or 100 nM) for 2 hr or 4 hr as indicated. Cells were fixed and stained with DCF-DA (10 μM) to show ROS-induced oxidation (green). The level and subcellular localization of endogenous γ-H2AX was detected using an antibody (Ab) against γ-H2AX, followed by an Alexa Fluor-594 (red)-conjugated secondary Ab, and analyzed with a confocal fluorescence microscope. DAPI was used to visualize the nuclei. Merged images show the co-localization of γ-H2AX and ROS production. Scale bar: 20 μm.

Supplemental Materials and Methods

Chemicals and antibodies

The BPA and E2 were purchased from Sigma, dissolved in ethanol (EtOH), and stored in aliquots at −20°C. The primary antibodies used for Western blot and immuno-fluorescence staining were specific to phospho-ATM (pS1981; Epitomics), ATM (Bethyl Laboratories), c-Myc (9E10; Roche Applied Science), γ-H2AX (pS139) and H2AX (EMD Millipore), ERα (Neomarkers, Thermo Scientific), cyclin D1, cyclin E, and BRCA1 (sc-642) (Santa Cruz Biotechnology), E2F1 (EMD Millipore), BRCA2 (Cell Signaling Technology), and β-Actin (Sigma). The dye 4'-6-Diamidino-2-phenylindole (DAPI) was obtained from Sigma. Horseradish peroxidase-conjugated secondary antibodies against rabbit, mouse, and goat were purchased from Jackson ImmunoResearch Laboratories and Alexa Fluor-488 (green)-conjugated secondary antibodies were obtained from Invitrogen, Life technologies.

Cell culture conditions

All cell lines were grown under normal conditions at 37°C and 5% CO_2. The MCF7 (ERα-positive) and MDA-MB-231 (ERα-negative) cell lines originate from human breast epithelial adenocarcinomas. MCF7 were grown in phenol-free DMEM/F12 medium (cellgro, Mediatech, Inc.), which was supplemented with L-glutamine (Gibco), gentamycine (TEKnova), Antibiotic-Antimycotic (A-A) solution (cellgro, Mediatech, Inc), and 5% Charcoal-treated fetal bovine serum (FBS) (Atlanta Biologicals, Inc.). MDA-MB-231 cells were grown in DMEM/F12 medium supplemented with L-glutamine, A-A solution, and 10% FBS. The MCF10A (ERα-negative) and 184A1 (ERα-negative) human cell lines are the immortalized benign and normal breast epithelial cell lines, respectively. MCF10A cells were cultured in DMEM/F12 medium

supplemented with human EGF (EMD Millipore), insulin (Sigma), hydrocortisone (Sigma), cholera toxin (EMD Millipore), L-glutamine, A-A solution, 5% FBS, and 5% Donor Horse Serum (Atlanta Biologicals, Inc.). 184A1 cells were cultured in MEGM medium supplemented with a SingleQuot Kit (Lonza Inc.), which contained human EGF, insulin, hydrocortisone, bovine pituitary extract, and gentamicin sulfate.
